# Development of a phased discharge planning for patients undergoing airway stenting: a Delphi study informed by the timing it right framework

**DOI:** 10.3389/fmed.2026.1851329

**Published:** 2026-09-22

**Authors:** Rong Zhang, Yanhong Wang, Lin Su, Xuejiao Deng, Xiaoyan Li, Jing Liu, Jing Wei, Liping Cui

**Affiliations:** 1 Department of Respiratory and Critical Care Medicine, Shanxi Bethune Hospital (Shanxi Academy of Medical Sciences), Tongji Shanxi Hospital, The Third Hospital of Shanxi Medical University, Taiyuan, Shanxi, China; 2 Department of Nursing, Shanxi Bethune Hospital (Shanxi Academy of Medical Sciences), Tongji Shanxi Hospital, The Third Hospital of Shanxi Medical University, Taiyuan, Shanxi, China

**Keywords:** airway stenosis, airway stenting, Delphi method, patient discharge planning, transitional care

## Abstract

**Objective:**

This study aimed to develop a dynamic, phased discharge planning intervention and optimize the nursing care pathway for patients with airway stenosis undergoing airway stenting, to address gaps in complication management and home care competencies following the procedure.

**Methods:**

Employing the Timing It Right (TIR) as a theoretical basis, the initial program was developed through integrated literature review, semi-structured interviews with 7 physicians, 9 nurses and 8 patients, and expert panel group discussions. Subsequent refinement involved three iterative rounds of Delphi consultation with domain experts. Revisions were made based on aggregated feedback to finalize the program.

**Results:**

Three rounds of expert consultation were completed, with questionnaires distributed via email and WeChat. Response rates were 95.24, 100, and 100% across the respective rounds. The expert authority coefficient (Cr) exceeded 0.7 throughout; coefficient of variation ranged from 0.05 to 0.17, 0.06 to 0.23, and 0.06 to 0.19; Kendall’s W values were 0.172, 0.166, and 0.187, respectively (*p* < 0.001). The final discharge plan comprised 5 primary domains: diagnosis phase (admission to pre-surgery), stabilization phase (postoperative to stable recovery), preparation phase (3 days pre-discharge), implementation phase (post-discharge to 1 month), adaptation phase (1–3 months post-discharge), plus 15 secondary categories and 57 tertiary items. All 57 tertiary indicators satisfied the retention criteria of mean importance score >4.00 and coefficient of variation <0.25 after three rounds of revision.

**Conclusion:**

The discharge planning intervention, structured around the Timing It Right Framework, demonstrates scientific rigor and operational feasibility, offering a standardized and comprehensive roadmap for the post-stenting care of patients. Prospective clinical trials are required in future research to quantify its effects on complication incidence, unplanned readmission and patient quality of life.

## 1 Background

Airway stenosis arises from diverse etiologies, including tumor-induced compression and granulation tissue proliferation following endotracheal intubation, typically presenting as exertional dyspnea. Progressive narrowing may culminate in life-threatening dyspnea or fatal asphyxia (1). Pharmacological interventions offer limited efficacy in addressing structural stenoses, and surgical approaches, though definitive, are invasive and poorly tolerated by certain patient populations. In this context, airway stenting presents a minimally invasive alternative with distinct clinical benefits (2). This technique entails the deployment of a tubular tracheal stent into the stenotic segment under bronchoscopic or fluoroscopic guidance, thereby mechanically restoring airway patency and alleviating dyspnea symptoms (3, 4).

However, airway stenting is not without risks. Postoperative complications are common and significantly impact patient outcomes: stent migration occurs in 8–25% of cases, often requiring reintervention (5); infection, including pneumonia and airway colonization, affects 15–30% of patients, prolonging hospital stays and increasing mortality risk (6); restenosis due to granulation tissue hyperplasia develops in 20–40% of individuals within 6–12 months, leading to recurrent dyspnea and readmissions (4). A multicenter study by Liu et al. reported that 32% of patients with malignant airway obstruction experienced at least one major complication within 3 months of stenting, with 18% requiring emergency revisional procedures (4). These complications not only diminish quality of life but also impose substantial economic burdens, with average reintervention costs exceeding $10,000 per episode in developed healthcare systems (7).

Following airway stent placement, partial coverage of the airway mucosa disrupts ciliary function, impairing mucociliary clearance. Inadequate care post-procedure may result in mucus retention, infection, secretion obstruction, mucosal perforation, and potentially life-threatening asphyxia. Patients must acquire and adapt disease-related knowledge and nursing competencies throughout all stages—from acute onset and stent implantation to at-home recovery. Nevertheless, the evolving implementation of enhanced recovery protocols mandates a reduced average length of hospital stay. Given the complexity of airway stenting as an interventional respiratory procedure, patients frequently lack sufficient time to assimilate necessary self-care strategies during hospitalization. Clinical records from our hospital in 2024 indicated an 18% rate of unplanned readmissions during home convalescence due to restenosis, stent migration, infection, and related complications, primarily attributed to inadequate home care capabilities. This highlights the pressing need to refine comprehensive, phase-specific patient management strategies.

Existing discharge guidance for airway stenting patients suffers from several limitations: it is typically delivered as a one-time, standardized instruction focused on medication adherence and basic wound care, lacking individualization for patient-specific risks (e.g., high-risk groups for migration) (8); it fails to address the dynamic nature of post-stent recovery, providing static advice that does not adapt to changing needs during the weeks to months following discharge (9); and it rarely integrates psychological support or family caregiver training, despite evidence that these factors strongly influence complication rates (10). These gaps contribute to the suboptimal transition from hospital to home care, as noted in a systematic review by Chen et al. (9), which found that conventional discharge strategies reduced readmissions by only 5–8% in respiratory intervention patients—far below the 30% reduction achievable with structured transitional care (9).

Discharge planning service, distinct from traditional one-size-fits-all discharge guidance, refers to a systematic, multidisciplinary intervention initiated early during hospitalization. It involves personalized assessment of patient and caregiver needs, development of phase-specific care protocols, and coordinated follow-up across healthcare settings to ensure continuity (11). Unlike standard discharge instructions, which are often documentation-focused and delivered shortly before departure, discharge planning services emphasize proactive risk stratification, skill-building for self-management, and linkage to community resources—elements shown to reduce 30-day readmission rates by up to 40% in chronic disease populations (12).

The Timing It Right (TIR) Framework, developed by Cameron et al. (11), delineates the trajectory of illness into five sequential phases, each with unique care priorities that align closely with the rehabilitation trajectory of airway stenting patients:

(1) Event/diagnosis phase: Encompasses the period from admission to pre-surgery, focusing on education about airway stenosis and stent procedures, as patients and families grapple with the diagnosis and treatment decisions.
(2) Stabilization phase: Occurs in the immediate postoperative period (1–7 days), prioritizing acute complication prevention (e.g., stent migration, bleeding) and physiological adaptation to the stent.
(3) Preparation phase: Defined as 3 days prior to expected discharge from the postoperative stabilization ward, rather than a fixed postoperative day count. The 3-day window was selected for two clinical reasons: First, most patients with airway stenting achieve stable vital signs, relieved dyspnea and controlled acute complications within 4–7 postoperative days; a 3-day advance window reserves sufficient time for systematic home care training, emergency skill teaching and caregiver assessment. Second, even for patients with prolonged hospitalization due to poor recovery, the 3-day pre-discharge unified standard avoids inconsistent training duration caused by variable hospital stays, and ensures standardized home care capacity assessment before hospital discharge. It emphasizes training in home care skills (e.g., airway humidification, symptom monitoring) and ensuring caregiver readiness.
(4) Implementation phase: First month post-discharge, focusing on reinforcing self-management behaviors, addressing early home care challenges, and adjusting interventions based on real-world performance.
(5) Adaptation phase: 1–3 months post-discharge, aiming to consolidate long-term self-efficacy, integrate patients into community care networks, and monitor for late complications (e.g., restenosis).

When integrated with the TIR framework, discharge planning service offers refined guidance for optimizing rehabilitation pathways, enhancing nursing productivity, and allocating medical resources more effectively by systematically pinpointing and addressing the “critical moments” within the clinical course of airway stenting. This study therefore developed a targeted discharge planning intervention tailored to airway stenting patients, grounded in the TIR framework, to enhance therapeutic outcomes and streamline the transition from hospital to home.

The core research questions were: (1) Based on the Timing It Right framework, what staged core components and specific indicators should be included in discharge planning for patients receiving airway stenting? (2) Through three rounds of Delphi expert consultation, can a scientific, operable staged discharge planning scheme covering the whole perioperative and post-discharge rehabilitation cycle be constructed? (3) What are the clinical implementation suggestions and follow-up optimization directions of the staged discharge planning scheme for airway stent patients?

## 2 Methods

### 2.1 Establishment of a research team

The research group comprised eight professionals: one head nurse from the Department of Respiratory and Critical Care Medicine, two deputy chief physicians with expertise in airway stenting, two critical care nurses, one respiratory therapist, two graduate students specializing in nursing, and one medical statistician who independently completed all Delphi statistical calculations and verified analysis results with a third-party hospital statistician. The head nurse acted as the coordinator, overseeing organizational and collaborative functions. Core responsibilities included conducting a literature review, performing semi-structured interviews, and holding group discussions to produce the initial program draft. Additional duties involved expert selection, questionnaire design for expert consultation, data collection through consultation rounds, synthesis and revision of expert feedback, and subsequent statistical analysis. The study adhered to the Declaration of Helsinki and received ethical approval from the institutional Ethics Committee (YXLL-2025-051). Written informed consent was obtained from all participants.

### 2.2 Drafting of the program

#### 2.2.1 Literature search

A comprehensive search strategy was implemented using the following terms: “airway stenosis/bronchial stenosis/respiratory stenosis,” “stenting/stenting therapy/stent intervention/airway stenting/bronchial stenting,” “discharge planning service/discharge planning/continuation of care/transitional care,” “Airway stenosis/Airway narrowing/Airway stricture/Bronchial stenosis/Bronchostenosis,” “Stent placement/Stent implantation/Stenting/Stent deployment/Stent insertion/Airway stenting/Bronchial stent implantation,” and “discharge/discharge planning/patient transfer/discharge coordination/discharge program/discharge service/discharge preparation/discharge intervention/discharge procedure/discharge instruction/discharge readiness/discharge guidance/continuity of patient care.” The databases searched included UpToDate, BMJ Best Practice, Guidelines International Network, Scottish Intercollegiate Guidelines Network, Registered Nurses’ Association of Ontario, National Institute for Health and Care Excellence, Australian Commission on Safety and Quality in Health Care, Medlive, PubMed, EMbase, Web of Science, the Cochrane Library, CINAHL, SinoMed, CNKI, Wanfang Medical Database, and VIP Database. The search included literature from each database’s inception through December 2024.

Inclusion criteria included studies involving patients with airway stenosis receiving stenting procedures, focusing on perioperative nursing care, published in either Chinese or English, and categorized as guidelines, evidence summaries, systematic reviews, expert consensus, or randomized controlled trials. Studies were excluded if the full text was unavailable or if the work constituted duplicate publication. Literature quality was appraised in accordance with the evidence grading and quality assessment framework established by the JBI Center for Evidence-Based Healthcare. Discrepancies among reviewers were resolved through arbitration by respiratory interventional therapy specialists. An initial retrieval yielded 1,931 records. Following title, abstract, and full-text screening, studies lacking full text, irrelevant content, or duplication were removed, resulting in the inclusion of 11 documents: 1 guideline (13), 4 expert consensus papers (14–17), 2 systematic reviews (18, 19), and 4 original research articles (6, 8, 20). Synthesizing the literature findings with clinical nursing practices and aligning with the conceptual model of the TIR framework, a preliminary framework was formulated to address the evolving care demands of airway stenting patients across different clinical phases.

#### 2.2.2 Semi-structured interviews

Based on literature analysis, a semi-structured interview approach was employed to iteratively refine and enrich the program. Participants—comprising physicians, nurses, and patients or their family members—were purposively selected from the respiratory and critical care department of a tertiary general hospital between February and April 2023. Data collection concluded upon thematic saturation, indicated by the absence of new topics.

Inclusion criteria for healthcare professionals were defined as follows: (1) physicians holding a master’s degree or higher with a minimum of 5 years’ clinical experience in diagnosing and managing airway stenosis through interventional procedures, or nurses possessing at least a bachelor’s degree and 10 or more years of professional practice in respiratory nursing; (2) all participants required an intermediate or higher professional title. Exclusion criteria included pregnancy, lactation, or any leave from clinical duties lasting 3 months or longer during the study period. The interview framework was structured around the following domains: (1) identification of primary clinical concerns and risk factors associated with discharge planning for patients undergoing airway stenting; (2) expert perspectives on staging the perioperative-to-discharge timeline and the corresponding variations in patient needs across these phases; (3) perceived barriers to the implementation of a phased-specific discharge planning service; and (4) professional recommendations for evaluating the effectiveness of such a phased intervention strategy.

Eligibility criteria included patients and their family members/caregivers included: (1) patients who had undergone airway stenting for stenosis and were in the stable recovery phase (≥7 days post-stenting, with no acute complications such as severe dyspnea, massive hemoptysis, or stent migration), or family members/caregivers who had been directly involved in the patient’s care for at least 5 days post-stenting; (2) voluntary participation and ability to communicate clearly.

Individuals who discontinued the interview process were excluded. The semi-structured interview outline comprised the following questions: (1) What was the primary concern during the stenting for you or your family member> (2) What challenges emerged throughout the treatment and nursing process> (3) What strategies were employed to address these difficulties> (4) What forms of support are considered beneficial in managing and overcoming these challenges>.

Prior to formal interviews, the interview outline underwent expert review for content validity: three experts (one chief respiratory physician, one clinical nursing specialist, and one methodologist with experience in qualitative research) evaluated the relevance, clarity, and comprehensiveness of the questions, resulting in revisions to 2 items (e.g., rephrasing “treatment process” to “perioperative and post-discharge care process” for specificity). A pilot test was then conducted with 2 patients and 1 caregiver to assess the feasibility of the interview process, leading to adjustments in question sequencing to improve flow.

Prior to the interviews, participants were scheduled in advance and informed of the interview’s objectives and relevance. To protect data integrity, all interviews were conducted in a quiet, controlled setting (department demonstration room), each lasting 15–30 min, with participants providing consent for full audio recording. After interviewing 7 physicians, 9 nurses, and 8 patients, thematic saturation was achieved; no novel insights emerged from the final 2 interviews, marking the endpoint of data collection. Audio recordings were transcribed within 24 h post-interview.

The Colaizzi method (21) was employed to analyze the data, following these steps: (1) familiarization with the data through repeated reading of transcripts; (2) extraction of significant statements related to discharge planning needs; (3) formulation of meanings from the significant statements; (4) clustering of formulated meanings into themes; (5) integration of themes into an exhaustive description of the phenomenon; (6) validation of the description against the original transcripts to ensure accuracy; (7) identification of fundamental structures of the phenomenon.

Dual independent coding was implemented for all interview transcripts: two graduate nursing researchers coded all significant statements separately without communication in advance. After independent coding was completed, the two coders compared coding results; the inter-coder reliability Kappa value was calculated as 0.86, indicating excellent consistency. All inconsistent coded themes were submitted to the research group and a qualitative research methodologist for joint discussion to reach unified coding conclusions. This process was used to structure and refine the preliminary framework for phased discharge planning services for airway stenting patients.

#### 2.2.3 Group meeting discussion

Based on the literature review and semi-structured interviews, an expert panel was convened to draft an initial version of the discharge planning service program for patients undergoing airway stenting. The panel comprised two chief respiratory physicians, one clinical nursing specialist, two respiratory nurses, and one respiratory therapist. Following iterative analysis of the proposed framework and its components, as well as revisions to specific elements, the finalized program included five primary domains, 15 secondary categories, and 57 tertiary items. Each component was accompanied by a comprehensive specification of implementation procedures.

### 2.3 Delphi expert consultation

#### 2.3.1 Drafting of the consultation questionnaire

The Delphi expert questionnaire comprised three main components: (1) Introduction section, presenting the rationale, objectives, and relevance of the questionnaire, along with detailed completion guidelines; (2) Tertiary item consultation form, delineating the specific components of the discharge planning service program for patients undergoing airway stenting. Each item was rated by experts using a 5-point Likert scale to assess its perceived importance, with the following definitions: 1 = “not important at all,” 2 = “slightly important,” 3 = “moderately important,” 4 = “very important,” 5 = “extremely important. A designated space was provided after each level 2 indicator for qualitative feedback; (3) Expert profile form, designed to gather demographic data—such as age, educational background, and professional title—alongside assessments of the expert’s authority, domain-specific expertise, and criteria used for professional judgment in the consultation process.

The first-round Delphi questionnaire contained two parts: (1) pre-set primary domains, secondary categories and tertiary items derived from literature analysis and semi-structured interview themes for experts to score importance; (2) blank open-ended comment columns under each secondary category and a global open-text box at the end of the questionnaire, allowing experts to supplement missing items, delete unreasonable indicators and adjust classification logic freely. All supplementary opinions proposed by experts in open-ended sections were sorted and integrated into the revised questionnaire for Round 2.

Prior to formal distribution, the questionnaire underwent pre-testing for reliability and validity: 10 experts (not included in the formal consultation) were invited to complete the questionnaire, and the content validity index (S-CVI = 0.92, I-CVI range = 0.85–1.00), indicating good content validity. Cronbach’s *α* coefficient for the importance ratings was 0.87, demonstrating acceptable internal consistency. Revisions were made to 3 items based on pre-test feedback (e.g., clarifying “home care” as “daily care activities such as airway humidification and symptom monitoring”).

#### 2.3.2 Expert selection

A purposive sampling strategy was employed to recruit 20 experts from 13 tertiary Class A general hospitals located in Shenzhen, Beijing, Shanghai, Wuhan, Xi’an, Chengdu, Zhengzhou, and Taiyuan. Eligibility criteria required over 10 years of clinical experience in respiratory medicine or nursing, a professional title at or above the intermediate level, substantial expertise in respiratory disease management or nursing, and provision of informed consent with voluntary participation in the consultation process.

During expert recruitment, we issued recruitment notices to 28 interventional pulmonology specialists and 11 respiratory rehabilitation therapists in national tertiary hospitals via respiratory intervention associations; only 1 rehabilitation therapist met the 10-year clinical experience and intermediate title inclusion criteria and agreed to participate, while all interventional pulmonologists declined due to heavy interventional surgery schedules and time constraints. Among the selected experts, age distribution was as follows: 5 aged 30–39, 7 aged 40–49, and 8 aged 50–59. Years of professional experience were distributed as follows: 2 with 5–9 years, 8 with 10–19 years, 5 with 21–30 years, and 5 with over 30 years. Educational backgrounds included 6 with bachelor’s degrees, 7 with master’s degrees, and 7 with doctoral degrees. Regarding professional rank, 9 held senior titles and 5 held associate senior titles.

#### 2.3.3 Expert consultation

Expert consultation was implemented via structured questionnaires distributed through Sojump (WJX) and printed formats. Participants assessed the importance and feasibility of each item and provided corresponding comments. Items scoring a mean above 4.00 with a coefficient of variation below 0.25 were subject to further evaluation by the research team, which determined their retention or elimination based on expert input (21, 22).

Each round of consultation was allotted a two-week response period, if participants failed to return the questionnaire by the deadline and did not request an extension, their responses were deemed unreturned and excluded from the analysis of that round.

Three iterative rounds were conducted. The criteria for expert consistency were defined as follows: Kendall’s W value (a measure of expert opinion concordance) with statistical significance (*p* < 0.05), and a coefficient of variation <0.25 for all items (indicating low variability in expert ratings).

Two objective stopping criteria were applied simultaneously to terminate Delphi consultation: (1) no new substantive revision suggestions or supplementary items were put forward by experts for two consecutive rounds; (2) all tertiary items met the screening threshold of coefficient of variation CV < 0.25; the original auxiliary standard of “the number of qualitative feedback decreased by more than 50% compared with the previous round” was only used as an auxiliary reference index rather than a core judgment basis. This study concluded when three pre-set criteria were met simultaneously: (1) the planned three rounds of consultation were completed; (2) expert consistency met the aforementioned standards (Kendall’s W significant, coefficient of variation <0.25); (3) auxiliary feedback saturation reference standard reached. This ensured high consistency across expert feedback and the scientific rigor of the final program.

After three rounds of revision and expert feedback integration, all final 57 tertiary items met the retention criteria of importance mean >4.00 and CV < 0.25; no indicators were retained beyond the threshold, and no items were eliminated during the consultation process.

### 2.4 Statistical methods

Data analysis was performed using SPSS 23.0. Descriptive statistics for expert demographics were presented as mean ± standard deviation and percentage composition. Indicators of expert engagement included effective response rate, valid questionnaire rate, and the proportion of constructive feedback. Expert authority (Cr) was quantified by combining judgment basis (Ca) and familiarity level (Cs) (23, 24). For missing data in the Delphi consultation questionnaires, the following strategies were adopted: (1) if the number of missing items for a single expert was ≤2, the mean value of the corresponding item ratings from other experts in the same round was used for imputation; (2) if the number of missing items exceeded 2, the questionnaire was excluded from analysis to ensure data reliability.

In Round 1, 1 returned questionnaire had 3 missing tertiary item scores and was directly excluded; 2 questionnaires had 1–2 missing items each, and mean imputation of the same item’s expert scores was adopted. Rounds 2 and 3 had zero missing data. The mean imputation method was selected because the missing items were scattered single indicators without concentrated missing of a whole dimension, which would not distort the overall expert scoring tendency. A post-hoc sensitivity analysis was conducted by comparing the ranking of item mean scores before and after imputation; the sorting order of all tertiary items remained unchanged, proving that mean imputation did not interfere with the overall Delphi results. Concordance among experts was measured using Kendall’s W, with higher W values indicating higher concordance. Statistical significance was defined as *p* < 0.05.

## 3 Results

### 3.1 Basic information of experts

Table 1 shows the demographic and professional characteristics of experts in the three Delphi consultation rounds. The expert group had 20 participants in Round 1 and 19 in Round 2and Round 3. In terms of age, 50–59-year-olds accounted for the highest proportion (40%), followed by 40–49-year-olds (35%) and 30–39-year-olds (25%), forming a mainly middle-aged, experienced team. For professional titles, senior (45%) and associate senior (25%) titles made up 70%, ensuring strong professional authority. Regarding specialty, nursing professionals were the most represented (65%), supplemented by respiratory medicine experts (30%) and 1 respiratory therapy specialist (5%), covering core disciplines of airway stenting care. In education level, master’s and doctoral degrees each accounted for 35% (total 70%), with bachelor’s degrees at 30%, indicating high educational attainment. For working experience, 10–19-year experience group was the largest (40%), while those with ≥20 years of experience accounted for 50% (25% with 20–29 years, 25% with ≥30 years), showing most experts had long-term clinical experience in the field.

**Table 1:** Basic information of experts consulted.

Item	Round 1 (*n* = 20)		Round 2 (*n* = 19)		Round 3 (*n* = 19)	
	*n*	Proportion (%)	*n*	Proportion (%)	*n*	Proportion (%)
Age (years)						
30 ~ 39	5	25	5	26.32	5	26.32
40 ~ 49	7	35	7	36.84	7	36.84
50 ~ 59	8	40	7	36.84	7	36.84
Professional title						
Intermediate	6	30	6	31.58	6	31.58
Associate senior	5	25	5	26.32	5	26.32
Senior	9	45	8	42.11	8	42.11
Specialty						
Respiratory medicine	6	30	6	31.58	6	31.58
Nursing	13	65	12	63.16	12	63.16
Respiratory therapy	1	5	1	5.26	1	5.26
Educational level						
Bachelor’s degree	6	30	6	31.58	6	31.58
Master’s degree	7	35	7	36.84	7	36.84
Doctoral degree	7	35	6	31.58	6	31.58
Working experience (years)						
5 ~ 9	2	10	2	10.53	2	10.53
10 ~ 19	8	40	8	42.11	8	42.11
20 ~ 29	5	25	4	21.05	4	21.05
≥30	5	25	5	26.32	5	26.32

### 3.2 Expert engagement

Across three rounds of consultation, expert engagement—as reflected in questionnaire response rates—was recorded at 95.24, 100, and 100%, respectively, with all returned questionnaires deemed valid. The 4.76% non-response rate in the first round was attributed to one expert’s temporary withdrawal due to unexpected participation in an urgent clinical conference, who was unable to complete and return the questionnaire within the specified two-week response period despite being reminded; this expert did not participate in subsequent consultation rounds. During the initial round, 85% of the experts (17 individuals) provided feedback; in the second round, this figure declined to 25% (5 experts), indicating consistently high participation levels throughout the consultation process.

### 3.3 Expert authority

Expert authority was quantified using the formula: Authority coefficient = (Cs + Ca)/2, where Cs represented the expert’s familiarity with the item and Ca denoted the basis of their judgment. According to the criteria proposed by Zhang et al. (25), an authority coefficient >0.7 indicates high expert authority. As shown in Table 2, all calculated authority coefficients exceeded 0.700, reflecting a consistently high level of authority among consulted experts.

**Table 2:** Expert authority coefficient.

Round	Familiarity coefficient (Cs)	Judgment coefficient (Ca)	Authority coefficient (Cr)
Round 1	0.893	0.820	0.856
Round 2	0.887	0.870	0.878
Round 3	0.894	0.880	0.887

### 3.4 Concordance of expert opinions

Kendall’s W across the three consultation rounds measured 0.172, 0.166, and 0.187, respectively, all with *p* < 0.001. Overall Kendall’s W values across the three rounds were 0.252 (Round 1), 0.214 (Round 2), and 0.220 (Round 3), suggesting a statistically significant trend toward concordance in expert opinions. Detailed results were presented in Table 3.

**Table 3:** Concordance coefficient of expert opinions.

Item level	Round 1			Round 2			Round 3		
	Kendall’s W value	χ^2^ value	*p*-value	Kendall’s W value	χ^2^ value	*p*-value	Kendall’s W value	χ^2^ value	*p*-value
Primary domain	0.256	20.444	<0.001	0.271	21.684	<0.001	0.304	24.314	<0.001
Secondary categories	0.329	91.99	<0.001	0.206	57.602	<0.001	0.168	47.052	<0.001
Tertiary items	0.172	192.433	<0.001	0.166	185.803	<0.001	0.187	208.901	<0.001
Overall (weighted)	0.252	284.867	<0.001	0.214	265.089	<0.001	0.220	280.267	<0.001

### 3.5 Item refinement results

Based on suggestions gathered across three Delphi consultation rounds and aligned with clinical practice realities, multiple revisions were implemented through structured team discussions.

Representative controversial qualitative comments from Round 1 experts were summarized as follows for transparency:

Rehabilitation therapist participation: Expert 12 (respiratory physician): “Rehabilitation assessment can be postponed to post-discharge adaptation phase; routine rehabilitation intervention is unnecessary in acute stabilization stage.” Expert 17 (specialist nurse): “Early respiratory rehabilitation training can reduce sputum retention, rehabilitation therapists should join multi-disciplinary team in diagnosis phase.”

Home follow-up frequency: Expert 5: “Weekly follow-up is too frequent for stable patients, biweekly follow-up is enough.” Expert 14: “Newly discharged patients face high complication risks, weekly follow-up is the minimum standard.”

Simulated stent teaching videos: Expert 3: “Complex mechanical principle videos will increase patient anxiety, simple picture brochures are better.” Expert 19: “Visual simulation videos improve patients’ understanding of stent migration risks.”

Qualitative feedback from experts highlighted several contentious items in the first round: (1) “Involvement of rehabilitation therapists in the discharge planning team” (coefficient of variation = 0.24), with debates on whether rehabilitation intervention was necessary during the acute phase; (2) “Frequency of home follow-up” (coefficient of variation = 0.23), with opinions divided between once weekly and twice weekly; (3) “Use of simulated videos for stenting principles” (coefficient of variation = 0.22), with concerns about patient comprehension of technical content.

For items with relatively wide expert disagreement during early revision, the research team adopted the following strategies: (1) Items with different expert rationales (e.g., “Follow-up frequency”) were revised to “tailored to individual needs with a minimum of once weekly” to balance expert opinions; (2) Items with unclear operational definitions (e.g., “rehabilitation therapist involvement”) were supplemented with specific scenarios to enhance feasibility; (3) All retained items met the pre-specified statistical thresholds after revision and were considered clinically relevant by the research team.

To better reflect the needs characteristic of the “diagnosis phase,” the composition of the discharge planning team was revised from “attending physicians, specialist nursing staff, nutritionists, and psychologists” to include “rehabilitation therapists” as well. The directive “Jointly communicate with patients on information needs and care needs during hospitalization” was revised to “Information support.” Additional items were introduced, including: “Provide a simulated version of the principle video,” “Guide effective coughing and expectoration, pursed lip breathing, abdominal breathing,” “Strengthen family emotional support for patients,” “Complications observation and care,” “Selection of humidified fluid for home care,” and “Wear a mask when going out, reduce visits to crowded places, and avoid upper respiratory tract infections.” During the “stabilization phase,” technical specifications related to stent adaptability management were further delineated. For the “preparation phase,” emphasis was placed on enabling patients to assess stent condition and respond to emergencies during home recovery. Within the “implementation” and “adaptation” phases, follow-up interventions were aimed at reinforcing patient and caregiver self-management capabilities, alongside the inclusion of “recommendation of authoritative health education platforms to patients and their families.” The coefficient of variation across the three Delphi rounds ranged from 0.05–0.17, 0.06–0.23, and 0.06–0.19, all remaining below the 0.25 threshold, indicating substantial concordance among experts. Ultimately, a comprehensive discharge planning framework for airway stenting patients was finalized, comprising 5 primary domains, 15 secondary categories, and 57 tertiary items. The detailed indicators of the phased discharge planning pathway and home-based follow-up and self-management pathway are fully summarized in Table 4.

**Table 4:** Discharge planning program for patients undergoing airway stenting based on Timing it Right Framework.

Intervention plan	Mean	Standard deviation(S)	Coefficient of variation (CV)
1. Diagnosis phase (from admission to pre-surgery)	4.2500	0.4443	0.1045
1.1 Information Support	4.6500	0.4894	0.1052
1.1.1 A multidisciplinary discharge planning team composed of the attending physician, specialist nurse, rehabilitation therapist, nutritionist, and psychologist is established.	4.2500	0.4443	0.1045
1.1.2 Information and care needs are assessed through direct communication with the patient during hospitalization.	4.6000	0.5026	0.1093
1.1.3 The patient and family receive detailed explanations regarding the etiology, risk factors, clinical presentation, and potential complications associated with airway stenosis.	4.3000	0.4702	0.1093
1.1.4 Surgical procedures, associated risks, anticipated recovery timelines, and complications related to airway stenting are clarified through verbal instruction and an educational video using a human simulator.	4.3500	0.4894	0.1125
1.2 Technical Requirements	4.3500	0.4894	0.1125
1.2.1 Adherence to prescribed oxygen therapy protocols is ensured, along with consistent monitoring and supportive care.	4.3500	0.4894	0.1125
1.2.2 Patients are instructed to assume semi-recumbent or sitting positions to alleviate dyspnea.	4.5000	0.5130	0.1140
1.2.3 Breathing techniques such as pursed-lip and abdominal breathing are introduced to ease respiratory effort and facilitate sputum clearance, along with training in effective coughing and expectoration.	4.8000	0.4104	0.0855
1.2.4 Adequate hydration is maintained to reduce thick sputum viscosity and enhance airway clearance.	4.2000	0.4104	0.0977
1.3 Emotional Support	4.8500	0.3664	0.0755
1.3.1 Therapeutic rapport is cultivated to foster patient trust and promote effective communication.	4.0500	0.6863	0.1695
1.3.2 Patient concerns are addressed promptly to mitigate emotional distress.	4.5500	0.5104	0.1122
1.3.3 Psychological support is extended to family members to enhance their capacity to provide emotional reinforcement.	4.3000	0.4702	0.1093
1.3.4 Referrals to licensed psychotherapists are made as necessary for specialized intervention.	4.2000	0.6156	0.1466
2. Stabilization Phase (Postoperative to Stable Recovery)	4.2500	0.4443	0.1045
2.1 Information Support	4.2000	0.6156	0.1466
2.1.1 Post-discharge care plans, including pharmacologic regimens, dietary guidance, and exercise protocols, are clearly communicated.	4.0500	0.7592	0.1874
2.2 Technical Requirements	4.2000	0.4104	0.0977
2.2.1 Within the first postoperative week, severe coughing and intense physical activity are avoided to minimize the risk of stent migration; antitussive agents are administered as indicated. Instruction in effective coughing and sputum clearance is continued.	4.2000	0.8335	0.1985
2.2.2 Airway humidification via nebulization is scheduled 4–6 times daily to maintain mucosal moisture and prevent sputum thickening.	4.5000	0.5130	0.1140
2.2.3 Suctioning is administered as indicated, with shallow suctioning depth (8–10 cm).	4.3500	0.6708	0.1542
2.2.4 Abdominal and deep breathing exercises are encouraged to improve respiratory muscle strength and vital capacity.	4.5000	0.5130	0.1140
2.2.5 Patients receive clear instructions regarding medication regimens, including dosage, potential adverse effects, and appropriate management strategies.	4.3500	0.4894	0.1125
2.2.6 Pain should be promptly reported to healthcare personnel; non-pharmacologic interventions such as music, meditation, or cognitive distraction are recommended. For pain scores ≥4, medication is administered in accordance with medical guidance.	4.7500	0.4443	0.0935
2.2.7 Nutritional intake focuses on light, digestible, and nutrient-rich foods, while avoiding spicy, oily, or irritant items to minimize airway stimulation.	4.2000	0.7678	0.1828
2.2.8 Daily fluid intake is maintained at ≥1,500 mL to aid in sputum clearance and optimize drug clearance.	4.7000	0.5712	0.1215
2.2.9 Routine oral care is implemented to reduce microbial colonization.	4.3000	0.8013	0.1864
2.2.10 Patients are educated on the early signs of complications—such as bloody phlegm or pharyngeal discomfort—and trained to report these symptoms without delay.	4.3500	0.7452	0.1713
2.3 Emotional Support	4.5500	0.5104	0.1122
2.3.1 Psychological distress is mitigated through guided techniques including deep breathing, music therapy, mindfulness, distraction, and social interaction.	4.4500	0.5104	0.1147
2.3.2 Caregivers are instructed to actively support patients’ rehabilitation and personal care, while maintaining effective supervision.	4.5500	0.5104	0.1122
3. Preparation Phase (Three Days Prior to Discharge)	4.4500	0.5104	0.1147
3.1 Information Support	4.3500	0.6708	0.1542
3.1.1 Family members or caregivers are contacted to confirm readiness for discharge, including the availability of required items, transportation logistics, and the suitability of the home environment.	4.2000	0.5232	0.1246
3.1.2 Patients and families are briefed on emergency response procedures post-discharge—such as managing airway restenosis, hemorrhage, or stent migration—through immediate oxygen administration, airway clearance, and emergency contact activation.	4.6500	0.6708	0.1443
3.1.3 A WeChat group is established to disseminate information on community healthcare and follow-up services, supporting reintegration into daily life and enhancing home-based care and follow-up continuity.	4.0000	0.5620	0.1405
3.2 Technical Requirements	4.4500	0.5104	0.1147
3.2.1 Airway humidification solutions are prepared using a 1:1 mixture of 0.9% sodium chloride injection and sterile water (or purified drinking water), with a volume of 5–10 mL per administration.	4.2000	0.5232	0.1246
3.2.2 Nebulization is performed 2–3 times daily, each session lasting 15–20 min, to maintain mucosal hydration and reduce thick sputum viscosity.	4.3500	0.5871	0.1350
3.2.3 Patients are instructed in effective coughing and sputum expectoration techniques to facilitate mucus clearance and prevent stent obstruction.	4.3000	0.6570	0.1528
3.2.4 Breathing exercises involve deep inhalation followed by controlled exhalation to soothe throat irritation and suppress excessive coughing; gentle pressure on the throat or abdomen may assist in controlling the cough reflex.	4.3000	0.7327	0.1704
3.2.5 Patients and caregivers are trained to monitor clinical signs, including sputum characteristics (color, volume, character), presence of irritating cough, chest discomfort, hemoptysis, and any symptom progression.	4.2000	0.7678	0.1828
3.2.6 A structured “home diary” is maintained to track daily respiratory rate, sputum output and characters, activity levels, dietary intake, and medication adherence.	4.4000	0.5982	0.1360
3.2.7 When outside the home, wearing a mask is essential; exposure to crowded environments is minimized to reduce upper respiratory tract infection risk.	4.1500	0.4894	0.1179
3.3 Emotional Support	4.3000	0.5712	0.1328
3.3.1 Recognition of the patient’s efforts during hospitalization is emphasized to reinforce recovery confidence.	4.2000	0.4104	0.0977
3.3.2 Family members and caregivers receive positive feedback on their care practices, fostering mutual support and enhancing caregiving confidence.	4.4000	0.5982	0.1360
4. Implementation Phase (Post-Discharge to One Month)	4.9000	0.3078	0.0628
4.1 Information Support	4.3500	0.4894	0.1125
4.1.1 Follow-ups are tailored to individual needs and conducted through telephone, WeChat, cloud-based platforms, outpatient visits, or community health sessions, with a minimum frequency of once per week.	4.5000	0.6070	0.1349
4.1.2 Ongoing assessment of home care practices informs targeted support, including access to reliable resources on disease management and caregiving strategies.	4.3500	0.7452	0.1713
4.1.3 Patients and families are directed to verified health education platforms and encouraged to review successful bronchial stenting self-management cases to obtain practical knowledge and reinforce confidence.	4.3000	0.8013	0.1864
4.2 Technical Requirements	4.1500	0.3664	0.0883
4.2.1 A health-conscious lifestyle is promoted, avoiding exposure to tobacco smoke, air pollutants, and known allergens such as pollen and dust mites in sensitized individuals.	4.3500	0.5871	0.1350
4.2.2 Airway humidification continues under medical orders, with adjustments based on sputum characteristics to ensure optimal secretion clearance.	4.3000	0.5712	0.1328
4.2.3 Dietary Guidance: Adequate hydration is maintained, and the diet includes fresh fruits and vegetables to aid in clearing internal heat and alleviating cough symptoms. Spicy, greasy, and other irritant foods are avoided to minimize airway irritation.	4.1000	0.7881	0.1922
4.2.4 Fiber-rich foods such as legumes and vegetables are recommended to promote regular bowel movements and reduce the need for straining during defecation.	4.4500	0.6048	0.1359
4.2.5 Patients are advised to adjust clothing promptly based on environmental changes to avoid exposure to cold, thereby reducing the risk of respiratory tract infections.	4.7000	0.4702	0.1000
4.3 Emotional Support	4.3000	0.5712	0.1328
4.3.1 Patients are encouraged to express their concerns, with timely identification and redirection of negative emotional states.	4.1500	0.6708	0.1616
4.3.2 Active participation in WeChat group discussions is recommended to foster peer communication and mutual support.	4.2000	0.6959	0.1657
4.3.3 Recognition of caregivers’ efforts helps alleviate emotional burden and reinforces the caregiving relationship.	4.4500	0.6048	0.1359
5. Adaptation Phase (1–3 Months Post-Discharge)	4.6500	0.4894	0.1052
5.1 Information Support	4.8500	0.3664	0.0755
5.1.1 Biweekly follow-ups are conducted to monitor psychological well-being and address emerging challenges.	4.2000	0.7678	0.1828
5.1.2 Detailed information on community medical resources is provided to support reintegration into daily life.	4.2000	0.5232	0.1246
5.1.3 Regular examinations are recommended every 6–12 months. Patients are instructed to seek immediate medical evaluation in cases of suspected stent restenosis or acute massive airway hemorrhage.	4.0000	0.3244	0.0811
5.2 Technical Requirements	4.5000	0.6883	0.1529
5.2.1 Adjustments to the airway humidification regimen and sputum clearance techniques are guided by entries in the “home diary” and the patient’s evolving clinical status.	4.2000	0.5232	0.1246
5.2.2 A proper rest period of 1 to 3 months post-discharge is recommended, with a gradual return to routine non-strenuous occupational tasks; heavy physical labor should be strictly avoided.	4.8500	0.3664	0.0755
5.2.3 Aerobic activity is introduced progressively, ideally 30–60 min per session, performed once daily or 3–5 times weekly. High-intensity exercise, competitive sports, and breath-holding activities are contraindicated.	4.5500	0.5104	0.1122
5.2.4 Patients and caregivers are instructed to monitor for signs of long-term complications and to seek timely medical evaluation when adverse symptoms arise.	4.1000	0.3078	0.0751
5.3 Emotional Support	4.3000	0.8013	0.1864
5.3.1 Constructive feedback and acknowledgment of rehabilitation progress are provided to mitigate psychological distress and reinforce commitment to long-term recovery.	4.5500	0.5104	0.1122
5.3.2 Emotional support for family members and caregivers is addressed through timely information delivery and practical assistance aligned with their caregiving responsibilities.	4.3000	0.4702	0.1093

Supplementary Table S1 presents the full revision log of all tertiary items across three Delphi rounds, including each indicator’s Round 1–3 importance mean, CV value, specific revision content and corresponding expert feedback basis, which intuitively reflects the iterative optimization process of the staged discharge planning scheme. Supplementary Appendix 1 provides representative qualitative feedback excerpts from experts during Delphi consultation.

## 4 Discussion

### 4.1 Summary of core findings and multidimensional value of the discharge planning program

This study developed a phased discharge planning program for patients undergoing airway stenting using the Timing It Right (TIR) Framework, refined through three rounds of Delphi consultation with experts. The final program comprises 5 primary domains (diagnosis, stabilization, preparation, implementation, and adaptation), 15 secondary categories, and 57 tertiary items, covering information support, technical requirements, and emotional support tailored to each phase of post-stenting recovery.

Discharge planning has emerged as a key intervention for enhancing post-stenting rehabilitation outcomes and streamlining healthcare resource utilization, positioning it at the forefront of contemporary clinical nursing research. This study developed a structured discharge planning program tailored to patients’ evolving needs following airway stenting, using the TIR framework as the foundational model to implement phased, multidimensional support aimed at ensuring patient safety. During the initial “diagnosis phase,” collaborative input from a multidisciplinary team—including physicians, nurses, and psychologists—alongside active family involvement, established a coordinated “physician-nurse–patient-family” alliance. This integrated approach helped mitigate patient anxiety while reinforcing disease comprehension and treatment adherence. In the subsequent “stabilization phase,” the intervention emphasized acute complication prevention and addressed specific physiological discomforts associated with artificial airways characteristic of this phase. The intervention centered on the standardization of airway humidification and clearance protocols, supplemented by nutritional support and initiation of early-stage rehabilitation training. During the “preparation phase,” family members received targeted guidance on emergency interventions—such as oxygen administration and emergency contact procedures in the event of airway restenosis (item 3.1.2)—which may effectively reduce the self-care pressure of family caregivers. This aligns with Smith et al.’s conclusion that family support served as a determinant in reducing hospital readmissions (10). In the subsequent “implementation phase,” the implementation of a “home diary” enabled dynamic tracking of respiratory indicators, including respiratory rate and sputum characters (item 3.2.6), thereby enhancing patient self-monitoring capabilities. This approach reflects the principles of patient empowerment advocated in contemporary nursing frameworks (26). Empirical evidence demonstrates that active patient engagement in disease management correlates with reduced postoperative complications and improved adherence to therapeutic regimens (11). Furthermore, the “implementation phase” incorporated cloud-based follow-up and WeChat group interactions, enabling cross-temporal and spatial coordination of medical services. This model enhances nursing responsiveness through consistent remote interventions, such as a minimum of one weekly follow-up (item 4.1.1). Research indicates that digital tools contribute to reduced outpatient utilization and lower overall healthcare expenditures (26, 27).

The Kendall’s W values in this study (0.172, 0.166, and 0.187 across three rounds) indicate moderate concordance among experts, which may be attributed to several factors: (1) The interdisciplinary nature of the expert panel (including physicians, nurses, and respiratory therapists) may have led to divergent perspectives on prioritizing clinical versus nursing-focused interventions; (2) Variability in clinical practice patterns across different regions (e.g., Shenzhen vs. Taiyuan) could contribute to differences in expert opinions on follow-up frequency and home care protocols; (3) The complexity of airway stenting procedures—encompassing both benign and malignant etiologies—may have introduced heterogeneity in expert judgments regarding phase-specific interventions.

This moderate consistency is consistent with published Delphi studies on respiratory disease transitional care protocols. Li et al. (22) constructed a COPD continuous nursing scheme via Delphi, with tertiary item Kendall’s W = 0.153–0.191; a study by Zhang et al. (25) on emergency nursing standardized process reported W = 0.161–0.203. Moderate consistency is common in multi-disciplinary expert panels covering medicine, nursing and rehabilitation specialists, due to disciplinary differences in clinical priority judgment, regional medical resource gaps and different management strategies for benign/malignant airway stenosis. All W values in this study reached statistical significance *P* < 0.001, which verifies the overall consistent tendency of expert core viewpoints. Despite these limitations, the statistical significance of Kendall’s W (*p* < 0.001) and the iterative refinement of items through three rounds of consultation ensure that the final program remains clinically meaningful.

### 4.2 Scientific rigor, reliability, and practicality of the discharge planning program for airway stenting patients

The discharge planning program, grounded in the TIR framework, was developed through a multifaceted methodology including systematic literature analysis, semi-structured interviews, expert panel discussions, and iterative Delphi consultations. Its structure reflects methodological rigor, reliability, and clinical adaptability. Regarding scientific rigor, the foundation was established through comprehensive literature review and qualitative input from 7 physicians, 9 nurses, and 8 patients via semi-structured interviews. Thematic analysis employed Colaizzi’s phenomenological method with dual independent coding to ensure accurate extraction of individualized needs related to rehabilitation guidance and psychological support. These insights were synthesized in a panel of 6 respiratory specialists, followed by three rounds of Delphi consultation, yielding authority coefficients of 0.856, 0.878, and 0.887. The final framework delineates consensus-driven priorities across five patient trajectory phases—diagnosis, perioperative, preparation, implementation, and adaptation—including informational, technical, and emotional domains. In terms of reliability, the Delphi process incorporated anonymized feedback and multiple iterative rounds to enhance concordance. Expert opinions exhibited strong concordance (Kendall’s W test, *p* < 0.01), confirming the consistency and stability of the final discharge planning program. In terms of applicability, patient-specific variables in airway stenosis—such as etiology, disease severity, age, and physical status—are systematically integrated into discharge planning. Tailored interventions are developed based on individual clinical profiles, including rehabilitation protocols, dietary recommendations, and activity limitations (item 4.2), thereby aligning care strategies with patient-specific needs to enhance adherence and optimize recovery outcomes. The outlined interventions were designed with a high degree of feasibility, enabling seamless implementation by clinical staff in routine practice. Concurrently, the program offers structured guidance to patients and family caregivers, fostering proactive engagement in treatment and recovery processes and supporting timely convalescence.

Secondary categories showed a decreasing trend in Kendall’s W values across rounds (0.329 → 0.206 → 0.168), which may be attributed to two key factors. First, secondary categories serve as a “connecting layer” between primary domains (e.g., diagnosis phase, stabilization phase) and tertiary items (specific operational measures). In Round 1, experts reached relatively high initial consensus on secondary categories because their scope (e.g., “Information Support” under the diagnosis phase) aligned with broad clinical experience. However, as consultation progressed to Rounds 2 and 3, experts focused more on refining tertiary items—such as adjusting the frequency of home follow-up or defining the role of rehabilitation therapists—which led to iterative revisions of specific measures. These revisions indirectly triggered re-discussions on the boundaries and content coverage of corresponding secondary categories, introducing slight differences in expert judgments. Second, the interdisciplinary background of the expert panel (including 65% nursing professionals, 30% respiratory medicine experts, and 5% respiratory therapists) contributed to divergent perspectives on secondary categories. For example, nursing experts tended to emphasize secondary categories related to practical care (e.g., “Technical Requirements”), while respiratory medicine experts paid more attention to categories linked to clinical risk control (e.g., “Complication Observation”). As rounds advanced, such discipline-specific priorities became more prominent in discussions of secondary category details, resulting in a mild decline in consistency compared to the initial round. Despite this decrease, the Kendall’s W values for secondary categories remained statistically significant (*p* < 0.001) across all rounds, confirming that expert opinions still maintained effective consensus on the core framework of secondary categories.

### 4.3 Targeted features of the discharge planning program for airway stenting patients

The discharge planning program tailored for airway stenting patients addresses the complexities inherent in postoperative rehabilitation and the unique characteristics of the Chinese healthcare context. The conventional care model, which often prioritizes inpatient treatment while overlooking post-discharge continuity, contributes to diminished self-management capacity and elevated risk of complications. This program adopts a phased, multidimensional intervention framework to systematically resolve key challenges.

(1) Meeting postoperative recovery demands across stages, particularly in restoring physiological function. Patients post-stenting are susceptible to complications such as stent migration, thick sputum, and dyspnea. To address these, nursing priorities are delineated in alignment with the TIR framework. Within the perioperative phase (first week post-surgery), standardized protocols are established for nebulization humidification (4–6 sessions/day), shallow suctioning (depth of 8–10 cm), and guided cough control to mitigate stent migration (items 2.2.1–2.2.3), consistent with findings by Lee and Khoo (5). In the preparation phase, patients are instructed to monitor indicators including respiratory rate and sputum characters (items 3.2.4–3.2.6), thereby enabling healthcare professionals to adjust care regimens remotely based on patient-generated data.
(2) Culturally responsive adjustments are incorporated to align with the familial caregiving norms prevalent in Chinese society, where patients often depend on family support despite caregivers’ limited clinical knowledge. This intervention enhances family involvement during the implementation and adaptation phases by integrating structured engagement strategies. Furthermore, the emotional support module—including mindfulness practices and peer-based mutual assistance—reflects culturally specific psychological tendencies, such as the reluctance to verbalize illness-related distress. These non-invasive approaches aim to mitigate postoperative anxiety and are consistent with Johnson et al.’s findings on the positive impact of psychological interventions on the quality of life in patients with respiratory conditions (28, 29).
(3) Stratified adaptation addressing variability in primary medical resources. Resource disparities—particularly the limited availability of nebulizers and inadequate follow-up resources—are frequently encountered in China’s primary care settings.

For resource-limited primary medical settings lacking nebulizers, cloud follow-up platforms and professional rehabilitation equipment, the scheme can be adaptively simplified: (1) Replace remote video follow-up with regular telephone follow-up, print paper self-management manuals instead of electronic educational videos; (2) If household nebulization equipment is unavailable, increase oral warm water intake to 2,000 mL per day to substitute partial airway humidification function; (3) Transfer complex respiratory rehabilitation training to community nurses during regular door-to-door visits; (4) Simplify the home diary into a single-page paper symptom recording form to reduce the operation burden of patients. Such low-cost adjustments can maintain the core staged care logic of the TIR framework without relying on high-end digital medical resources. To address these constraints, the program integrates cloud-based follow-up with targeted community nurse training, leveraging remote support mechanisms to bridge existing service gaps and enhance operational feasibility under resource-limited conditions.

(4) Dynamic adjustment to patient-specific characteristics. While the program outlines standardized procedures, adaptive modifications remain essential to accommodate patient-specific variables such as age and baseline pulmonary function. For instance, antitussive medication must be tailored according to individual tolerance thresholds to prevent excessive suppression of the cough reflex. Similarly, exercise rehabilitation regimens—e.g., 30–60 min of daily aerobic training—should be calibrated progressively in line with each patient’s physical endurance. Incorporating AI-driven analysis of home diary data in future iterations may enable individualized risk prediction (30), thereby advancing the paradigm of nursing care from standardization to precision-based models.

## 5 Limitations

This program offers several strengths; however, limitations remain that warrant comprehensive consideration.

First, all recruited experts were from 13 tertiary Grade A general hospitals in China, without representatives from secondary hospitals, community health service centers or overseas medical institutions. Since discharge planning extends to home and primary community care, the scheme’s external generalization ability is limited in grassroots medical environments and foreign healthcare systems. In addition, the expert group structure is dominated by nursing staff (65%), with insufficient interventional pulmonology and rehabilitation therapist representatives, which may bias the scheme toward nursing operational indicators rather than interventional risk management. Future multi-center validation covering multi-level medical institutions is required.

Second, the staged intervention cycle of the scheme only covers 3 months after discharge, failing to monitor long-term adverse events such as stent restenosis, repeated interventional treatment and long-term quality of life changes occurring within 6–12 months after stent implantation. Longitudinal cohort follow-up lasting more than 1 year is needed to evaluate the long-term clinical benefit of the scheme.

Third, the scheme relies heavily on cloud follow-up platforms, WeChat group communication and video teaching resources, which require patients and caregivers to possess basic digital literacy and intelligent equipment access. Elderly patients with low education level and poor digital operation ability, as well as families without stable network conditions, cannot fully implement all remote follow-up measures, reducing the applicability of the scheme for vulnerable aging groups.

Fourth, the current unified scheme does not set differentiated care modules for benign and malignant airway stenosis patients. Malignant stenosis patients have higher tumor progression risk and poorer physical tolerance, while benign patients face long-term granulation hyperplasia risk; their self-management demands and complication monitoring focuses are heterogeneous, and subgroup stratified optimization needs to be carried out in subsequent research.

## 6 Conclusion

In summary, the discharge planning program developed in this study enhances post-stenting rehabilitation outcomes and improves care efficiency through structured, phased interventions guided by the TIR framework and supported by multidisciplinary collaboration. Its design is validated using the Delphi method, confirming both scientific rigor and practical applicability in clinical contexts.

The priority of subsequent research should be a single-center prospective pilot clinical trial: recruit airway stent patients and divide them into routine discharge guidance group and TIR-based staged discharge planning intervention group, to compare the 30-day unplanned readmission rate, postoperative complication incidence, patient self-management efficacy score and caregiver satisfaction between the two groups, verifying the actual clinical effect of the scheme. After preliminary effectiveness verification, carry out multi-center expansion validation including secondary hospitals and community medical institutions. Long-term optimization directions such as AI intelligent home monitoring system can be developed on the basis of confirmed clinical benefits, combined with medical insurance policy research to promote the popularization of remote follow-up services. Future research should explore broader implementation across diverse care settings, including primary hospitals and different patient populations with varying etiologies. Additional priorities involve integrating technological innovation by designing AI-enabled home monitoring systems capable of delivering real-time complication alerts, and advancing the inclusion of digital follow-up services within medical insurance schemes to alleviate patients’ financial burden.

## Data Availability

The original contributions presented in the study are included in the article/Supplementary material, further inquiries can be directed to the corresponding author.
