# Supplementary material for: Development of a phased discharge planning for patients undergoing airway stenting: a Delphi study informed by the timing it right framework
Source: Front Med (Lausanne). 2026 Sep 22;13:1851329. doi: 10.3389/fmed.2026.1851329 (PMC13638610; doi:10.3389/fmed.2026.1851329)
Supplement: Supplementary file 1 [file Supplementary_file_1.docx]

| **Topic** | **Representative feedback** |
| --- | --- |
| Rehabilitation therapist participation | Expert 12 considered that rehabilitation assessment could be postponed to the adaptation phase, whereas Expert 17 recommended early respiratory rehabilitation training to reduce sputum retention and improve self-management. |
| Home follow-up frequency | Expert 5 suggested biweekly follow-up for stable patients, while Expert 14 emphasized that newly discharged patients remain at high risk and should receive at least weekly follow-up. |
| Simulated teaching videos | Expert 3 noted that complex technical videos might increase anxiety, whereas Expert 19 supported simplified visual simulation to improve understanding of stent migration risk. |
| Humidification protocol | Experts requested more operational descriptions of humidification fluid, frequency, and symptom-based adjustment to improve feasibility in home settings. |
| Community and resource-limited settings | Experts suggested retaining telephone follow-up and paper home diaries as alternatives when cloud platforms or video teaching are unavailable. |
