# Supplementary material for: Development of a phased discharge planning for patients undergoing airway stenting: a Delphi study informed by the timing it right framework
Source: Front Med (Lausanne). 2026 Sep 22;13:1851329. doi: 10.3389/fmed.2026.1851329 (PMC13638610; doi:10.3389/fmed.2026.1851329)
Supplement: Supplementary file 2 [file Table_1.docx]

**Supplementary Table S1 Iterative revision log of tertiary indicators across three Delphi rounds**

| **Tertiary Item ID** | **Round1 Mean** | **Round1 CV** | **Round2 Mean** | **Round2 CV** | **Round3 Mean** | **Round3 CV** | **Modification content** | **Expert feedback basis** |
| --- | --- | --- | --- | --- | --- | --- | --- | --- |
| 1.1.1 | 3.82 | 0.240 | 4.16 | 0.152 | 4.25 | 0.1045 | Add rehabilitation therapist into multidisciplinary team | Physicians proposed lack of rehabilitation assessment in early stage |
| 3.1.1 | 3.91 | 0.231 | 4.12 | 0.147 | 4.20 | 0.1246 | Add home environment pre-assessment content | Caregivers reflected unsuitable home facilities hinder self-care |
| 4.1.1 | 3.88 | 0.228 | 4.31 | 0.161 | 4.50 | 0.1349 | Revise fixed weekly follow-up to individualized minimum once weekly | Experts disputed fixed frequency for stable patients |
| 1.1.4 | 3.94 | 0.221 | 4.22 | 0.138 | 4.35 | 0.1125 | Add simulated stent teaching video intervention | Some experts suggested visual teaching improves comprehension |
